# Effect of ethanol on flavor perception of Rum

**DOI:** 10.1002/fsn3.629

**Published:** 2018-03-26

**Authors:** Chelsea M. Ickes, Keith R. Cadwallader

**Affiliations:** ^1^ Department of Food Science and Human Nutrition University of Illinois Urbana IL USA

**Keywords:** alcoholic beverage, descriptive analysis, ethanol, flavor, rum

## Abstract

This is the first sensory study to evaluate the effects of ethanol concentration on flavor perception of distilled spirits. Dilution series of two rums (R1 and R2) were evaluated to gain insight into the effects of ethanol concentration on the flavor perception of distilled spirits. Rums were diluted 1:2 (v/v) either with pure water to a final alcohol by volume (ABV) of 20% (R1‐W and R2‐W) or with an aqueous 40% ABV solution (R1‐E and R2‐E). The later dilution accounted for the flavor dilution effect while keeping the ethanol concentration the same as the original liquors. Descriptive sensory analysis was conducted on both dilution series and the original rums. Twenty‐three attributes were evaluated consisting of eight aroma, four aroma‐by‐mouth, four mouthfeel, two taste, and five aftertaste terms. Results revealed 18 significant attributes for the R1 series. With the exception of silky mouthfeel, all attributes were rated highest in R1 and lowest in R1‐E. The R2 series contained sixteen significant attributes, all of which were rated higher in R2 compared with R2‐E. The flavor profiles of the original rums and those diluted with water were very similar, with the diluted rums generally having slightly lower attribute intensities. In contrast, the rums diluted with 40% ABV had significantly different flavor profiles than the original rums. Results indicate that diluting spirits with water may reduce the odor suppression effects of ethanol or enhance flavor release which appears to counteract the flavor dilution effect.

## 1 INTRODUCTION

Ethanol is arguably the most important component of alcoholic beverages, particularly distilled spirits. Alcoholic beverages span a wide range of alcohol concentrations from beers (3%–10% ABV) to distilled spirits (usually 40% ABV). Furthermore, ethanol concentration plays an important role in the flavor perception of alcoholic beverages. Molecular interactions, flavor partitioning, and sensory perceptions have all been shown to be affected by ethanol concentration.

In terms of the physicochemical properties of ethanol, the water–ethanol structure greatly changes as the system goes from 100% aqueous to 100% ethanolic (Cipiciani, Onori, & Savelli, 1988; Conner, Birkmyre, Paterson, & Piggott, 1998; D'Angelo, Onori, & Santucci, 1994a,b; Franks & Ives, 1966; Onori & Santucci, 1996; Parke & Birch, 1999; Petrillo, Onori, & Sacchetti, 1989; Wakisaka, Komatsu, & Usui, 2001). In 100% aqueous systems, the water molecules exist in short‐range hydrogen‐bonded structures that are highly fluid, continuously breaking and reforming hydrogen bonds (Franks & Ives, 1966). As the ethanol concentration initially increases, the ethanol molecules are monodispersed within the aqueous matrix (D'Angelo et al., 1994a,b; Conner et al., 1998). This behavior continues until the ethanol concentration reaches approximately 15% alcohol by volume (ABV). Above this concentration, ethanol molecules begin to form aggregates or micelles (Cipiciani et al., 1988; D'Angelo et al., 1994a,b; Onori & Santucci, 1996). Once the solution reaches an ethanol concentration of 57% ABV, a final structural shift in the water/ethanol matrix occurs where the water molecules become monodispersed within the ethanolic matrix (D'Angelo et al., 1994a,b).

Previous research on how ethanol concentration affects aroma and sensory perception in alcoholic beverages has mainly approached the problem from an analytical perspective. Additionally, studies have mainly focused on how ethanol affects the headspace concentration of volatiles in static systems. As the ethanol concentration of solutions increases, the headspace concentration has been shown to decrease (Athès, Pena‐Lillo, Bernard, Perez‐Correa, & Souchon, 2004; Aznar, Tsachaki, Linforth, Ferreira, & Taylor, 2004; Boothroyd, Linforth, & Cook, 2012; Conner et al., 1998; Escalona‐Buendia, Piggott, Conner, & Paterson, 1998; Tsachaki, Aznar, Linforth, & Taylor, 2006). The decrease in headspace concentration is typically attributed to an increase in the solubility of aroma compounds as the ethanol concentration increases. Ickes and Cadwallader (2017a, b) reviewed the current research on ethanol's effects on flavor perception, and their paper provides a more detailed evaluation of the analytical research that has been performed to date.

Overall, sensory research exploring the effects of ethanol concentration on the perception of alcoholic beverages is lacking. The majority of studies have evaluated ethanol's effect on wine or wine model systems (Escudero, Campo, Fariña, Cacho, & Ferreira, 2007; Goldner, Zamora, Di Leo, Gianninoto, & Bandoni, 2009; Guth, 1998; Jones, Gawel, Francis, & Waters, 2008; King, Dunn, & Heymann, 2013; Le Berre, Atanasova, Langlois, Etiévant, & Thomas‐Danguin, 2007). While these studies demonstrate that ethanol concentration can impact flavor perception, the alcohol content of wine is very different from that of distilled spirits.

To date, the only sensory study to include distilled spirits was carried out in the 1970s by Williams (1972). Williams dealcoholized five different alcoholic beverages and observed the sensory changed compared with the original beverage. The study showed that de‐ethanolized whiskey was perceived as drier and had less bite than the full alcohol whiskey. Aside from this work, no sensory research has been conducted to provide insights into how ethanol's concentration may affect flavor perception during the consumption of distilled spirits.

While some consumers will only drink distilled spirits neat, others typically dilute their drink before consumption. A common practice is to consume spirits on the rocks, where the ice both cools and dilutes the beverage. Some people insist that a small splash of water is needed to open up the flavor. Still others state that distilled spirits need to be diluted to ~23% ABV to get the best flavor perception of the beverage. This has been the traditional practice in the whiskey industry for years, and a common reason given for this practice is to reduce the pungency of the alcohol (Smith & Roskrow, 2012).

Descriptive analysis (DA) panels allow researchers to identify and quantitate the sensory differences between products (Lawless & Heymann, 1999a; Meilgaard, Civille, & Carr, 2007; Stone & Sidel, 1993). Panelists develop terms, corresponding definitions and references for the sensory attributes they perceive in the product for the modalities of aroma, aroma‐by‐mouth, taste, mouthfeel, and aftertaste. Panelists rate the determined references and then use those as anchor points on the scale when evaluating the samples. Panelist finally rates the product in individual booths for all the attributes that were previously identified. The collected data are then subjected to statistical analysis to evaluate the samples using methods such as analysis of variance and principle component analysis.

The goal of this study was to evaluate using a descriptive sensory analysis the effects of ethanol concentration on the perceived sensory attributes of distilled spirits (rum) by comparing the original rums to those diluted 1:2 (v/v) with water or 1:2 (v/v) with aqueous 40% ethanol. The 1:2 (v/v) dilution with water dilutes both the congeners and ethanol strength while the 1:2 dilution with 40% ethanol dilutes only the congeners without changing the strength of the ethanol. The different dilutions allow the changes that may be attributed to the change in ethanol concentration versus the change in congener concentration to be assessed. It was hypothesized that changes in ethanol concentration would significantly affect the perceived sensory attributes, especially the aroma, of the rums, causing the dilutions to have significantly different profiles from the original full strength rum.

## 2 MATERIALS AND METHODS

### 2.1 Sample Selection

Two commercially available rums, Diplomatica Reserva Exclusiva (Destilería Unidas S.A., Venezuela) and Ron Abuelo: Añejo 7 years (Varela Hermanos, Panama), designated as rum 1 (R1) and rum 2 (R2), respectively, were purchased at a local liquor store (Champaign, IL). Both rums had a reported ethanol concentration of 40% alcohol by volume (ABV). Mention of the brand name of these rums does not imply any research contact or sponsorship and is not for advertisement or endorsement purposes.

### 2.2 Sample and Sensory Reference Preparation

Two dilutions of each rum were prepared, a 1:2 (v/v) dilution with pure water (Ice Mountain 100% Natural Spring Water, Nestlé Waters North America, Stamford, CT) (R1‐W and R2‐W), and a 1:2 (v/v) dilution with 40% ethanol (R1‐E and R2‐E). The 40% ethanol solution was prepared by diluting 190 proof (95% ABV) ethanol (USP Grade, Decon Labs, Inc., King of Prussia, PA) with pure water. Prior to testing (no more than 1 hr), 20 mL of each sample was measured into a black‐tinted double old‐fashion glass (Threshold, Target, USA) and the glass covered with a glass petri dish.

Attribute references were prepared daily and placed into 2 or 4 oz. lidded plastic soufflé cups (Dart Container Corporation, Mason, MI) and labeled with the reference identity. References were prepared fresh (no more than 24 hr) prior to evaluation. A complete list of attributes, definitions, references, reference scores, and preparation procedures is shown in Table 1.

**Table 1:** List of final attributes, definitions, references, reference scores, and reference preparations determined for the descriptive analysis panel on ethanol's effect on the flavor perception of rums

Modality	Attribute	Definition	Reference	Reference Score	Preparation
Aroma	Alcohol	Aroma associated with ethanol	71 proof alcohol (Decon Labs, Inc.; King of Prussia, PA)	11.5	10 oz. of (125 mL water + 75 ml 190 alcohol) in 2 oz. cup
	Caramel	Aroma of caramelized sugar	Caramel syrup (The J.M. Smucker Company; Orrville, OH)	12.5	2 g in 2 oz. cup
	Dark fruit	Aroma associated with dried dark fruits	Prunes (Sunsweet Growers Inc.; Yuba City, CA)	14	0.4 g (~1/8) prune in a 2 oz. cup
	Maple	Aroma of maple syrup	Maple extract (McCormick & Co., Inc.; Hunt Valley, MD)	13.5	one teaspoon in 500 mL volumetric flask, dilute to volume, 10 mL in 2 oz. cup
	Roasted	Aroma of medium roasted malted barley	Brown‐roasted barley (The Country Malt Group; Castleford, West Yorkshire, UK)	15	0.2 g in a 2 oz. cup
	Toasted	A browned sweet aroma associated with toasted marshmallow	Toasted marshmallow (Jet Puffed, Kraft Foods Group; Northfield, IL)	13	preheat boiler in oven, cut marshmallow in 1/8's, toast for 30 seconds and place 1/8 marshmallow in 4 oz. cup
	Vanilla	Aroma of natural vanilla extract	Natural vanilla extract (McCormick & Co., Inc.; Hunt Valley, MD)	11	1/4 teaspoon in 500 mL volumetric flask, dilute to volume, 10 mL in 2 oz. cup
	Woody	Aroma of a wood barrel	Oak wood chips (LD Carlson Company; Kent, OH)	10	0.5 g in 2 oz. cup
Mouthfeel	Astringent	A drying sensation in the mouth associated with a high tannin wine	Overbrewed green tea (Lipton, Unilever; Englewood Cliffs, NJ)	12	steep one tea bag in 300 mL of boiling water for 5 min, place ~15 mL in a 2 oz cup
	Silky	An uninhibited flow of liquid over the tongue, with a smooth feeling in the mouth	Almond milk (Silk, WhiteWave Foods; Broomfield, CO)	13	~15 mL in a 2 oz. cup
	Slick	A smooth tongue coating	Glycerin (Heritage Store; Virginia Beach, VA)	13.5	20 g of glycerin + 60 g water, ~10 g in a 2 oz. cup
	Warming	The increase in temperature perception in the mouth as a result of alcohol concentration	71 proof alcohol (Decon Labs, Inc.; King of Prussia, PA)	14	10 oz. of (125 mL water + 75 ml 190 alcohol) in 2 oz. cup
Taste	Bitter	Taste associated with a caffeine solution	Caffeine solution (Fisher Scientific; Fair Lawn, NJ)	12.5	1 g caffeine in 500 mL of hot water, stir until dissolved, ~15 mL in each cup
	Sweet	Taste associated with a sucrose solution	Cane sugar solution (C&H, Domino Foods, Inc.; Yonkers, NY)	11.5	4 g in 200 mL of water, stir, ~15 mL per cup
Aroma‐by‐mouth	Alcohol	Aroma‐by‐mouth associated with 40% or greater alcohol	71 proof alcohol (Decon Labs, Inc.; King of Prussia, PA)	14.5	10 oz. of (125 mL water + 75 ml 190 alcohol) in 2 oz. cup
	Caramel	Aroma‐by‐mouth of caramelized sugar	Caramel syrup (The J.M. Smucker Company; Orrville, OH)	10.5	caramel solution, 10 g of caramel dissolved in 200 mL of water, ~10 mL in a 2 oz. cup. Make daily
	Maple	Aroma‐by‐mouth of maple syrup	Maple extract (McCormick & Co., Inc.; Hunt Valley, MD)	12	one teaspoon in 500‐mL volumetric flask, dilute to volume, 10 mL in 2 oz. cup
	Vanilla	Aroma‐by‐mouth of natural vanilla extract	Natural vanilla extract (McCormick & Co., Inc.; Hunt Valley, MD)	12.5	1/4 teaspoon in 500‐mL volumetric flask, dilute to volume, 10 mL in 2 oz. cup
	Woody	Aroma‐by‐mouth of a woody barrel	Oak wood chips (LD Carlson Company; Kent, OH)	10.5	0.5 g in 2 oz. cup
Aftertaste	Bitter	Aftertaste associated with a caffeine solution	Caffeine solution (Fisher Scientific; Fair Lawn, NJ)	12.5	1 g caffeine in 500 mL of hot water, stir until dissolved, ~15 mL in each cup
	Brown spice	Aftertaste associated with brown spices such as clove, and nutmeg	Ground nutmeg (McCormick & Co., Inc.; Hunt Valley, MD)	10	1 g in 600 mL of water, stir 5 min, filter, 10 mL in 2 oz. cup
	Plastic	Aftertaste associated with PVC plastic	Vinyl shower curtain (Maytex Mills, Inc.; China)	11	1 in × 1 in piece, place on tongue
	Vanilla	Aftertaste associated with natural vanilla extract	Natural vanilla extract (McCormick & Co., Inc.; Hunt Valley, MD)	10	1/4 teaspoon in 500‐mL volumetric flask, dilute to volume, 10 mL in 2 oz. cup

### 2.3 Panelists

All materials related to panelist recruitment and test design were approved by the Institutional Review Board (IRB) at the University of Illinois Urbana‐Champaign (IRB Protocol Number: 16854). Eight panelists, (four men and four women, age range 23–66 years) participated in the descriptive analysis panel. Panelists were selected based on interest, sensory acuity, and availability. Panelists were University of Illinois Students and members of the local community who all had 20 +  hr of previous sensory training with distilled spirits. Panelists were also required to present a valid form of identification at the screening to verify that they were over 21 years of age.

### 2.4 Test design

A hybrid of Qualitative Descriptive Analysis^®^ (Stone, 1992) and the Spectrum™ method (Meilgaard et al., 2007; Muñoz & Civille, 1992) was used (Ickes & Cadwallader, 2017a). Two rums were evaluated as a dilution series. At each session, panelists received one dilution series consisting of three samples: straight rum, a 1:2 dilution with 40% ABV ethanol, and a 1:2 dilution with water (e.g., R1, R1‐E and R1‐W, respectively). On the first day of the panel, the panelists were refreshed about the DA method to be used in the study. The first four sessions (1 hr each) consisted of term and reference generation, followed by reference refinement. Panelists were presented with a dilution series of samples labeled with random three‐digit codes and asked to generate the attributes they perceived in the rum samples for aroma, aroma‐by‐mouth, mouthfeel, taste, and aftertaste modalities. Panelists were provided with a rum flavor wheel (Ickes, Lee, & Cadwallader, 2017) to aid in term generation. Through group discussion, panelists identified the terms to be used, developed a precise definition of each attribute, and determined a corresponding reference.

After the terms and references were established, panelists then spent two sessions (1 hr each) determining the reference intensities of each attribute. Panelists were asked to scale the references based on a 15 point scale, where zero is no perception of a given attribute and 15 is the strongest perception of that attribute in the rum samples. Panelists then spent 6 days (1 hr each) practicing scoring the rums, using the references as anchor points for the scale to aid in panel uniformity. On one of the days, panelists conducted individual booth practice sessions in Bevier Hall on the University of Illinois at Urbana‐Champaign campus for one 30‐min session using the Compusense five (Version 5.0: Guelph ON, Canada) data acquisition system. Panelists were routinely provided with their scoring results from the previous day to help identify and correct for attributes they were rating inconsistently with the rest of the group.

The panel concluded with 2 days of individual booth testing. Panelists attended two 30‐min sessions per day, evaluating one dilution series of three samples at each session. Testing took place in a room with partitioned booths maintained at 22°C. Rum samples were presented in black‐tinted double old‐fashion glasses covered with glass petri dishes and labeled with random three‐digit codes. Sample presentation was randomized among all panelists. Samples were evaluated under red lighting to mask the color differences between the dilutions. Compusense five software was used to record panelists’ responses. Panelists were provided with a reference tray when they arrived and encouraged to evaluate all references before proceeding into the booth for testing. Panelists were free to leave the booth at any time to re‐evaluate a reference.

### 2.5 Statistical analysis

Statistical analysis of the data was performed using Statistical Analysis System (SAS)^®^ (Version 9.4, SAS Institute Inc., Cary, NC, USA). Analysis of variance (ANOVA) was conducted on each of the 23 attributes evaluated during the DA panel to determine the presence of overall significant differences (*p *<* *.05) using the PROC GLM function for variations within the dilutions, panelists, replications, and their corresponding interactions: dilution by panelist (D × P), dilution by replication (D × R), and replications by panelist (R × P). Each rum dilution series was analyzed separately. The calculated probabilities were compared with significance levels α = .05, .01, and .001. When significant D × P interactions existed, adjusted *F*‐ratios were calculated using Microsoft^®^ Excel^®^ 2016 (Version 16: Microsoft Corporation, Redmond, WA) by dividing the dilution mean square by the D × P interaction mean square and calculating the new probability using the F.DIST function. Fisher's least significant difference (LSD) test was conducted on all attributes determined as significant by ANOVA.

Principle component analysis (PCA) biplots were produced using SAS and Microsoft Excel to create a visual representation of the data to allow further examination of the relationship of the rums to individual attributes that characterized the samples. Pearson correlations were calculated using the same SAS software, with significance determined at α = .05, .01, and .001.

## 3 RESULTS AND DISCUSSION

This is the first sensory study to examine the effects of ethanol concentration on flavor perception in distilled beverages. Sensory descriptive analysis was performed to characterize the effect of ethanol concentration on flavor perception. A series of three samples was evaluated for each rum consisting of the straight rum (directly from the bottle, 40% ABV), a 1:2 (v/v) dilution with water (creating a 20% AVB sample) to mimic how samples are routinely evaluated in industry, and a 1:2 (v/v) dilution with 40% ethanol to account for the flavor dilution effect while allowing alcohol concentration to remain constant. Two different rums were evaluated to assess whether the effects were sample specific or possibly applicable to a wider range of distilled spirits. Additionally, samples were evaluated in black glasses and under red lights to reduce any bias that might be caused by differences in color between the dilutions.

The panelists generated 23 attributes to describe the two different dilution series. The generated terms, term definitions, selected references, reference scores, and reference preparations are shown in Table 1. Reference scores are an average of individual panelist's ratings. All panelists had previous training evaluating the sensory properties of distilled beverages.

Analysis of variance (ANOVA) was conducted on the dilution series for each rum (R1 and R2) separately for all 23 attributes identified and rated by the panelists. The results are shown in Tables 2 and 3. In general, sample replication was not a significant source of error (*p* > .05) for either the R1 dilution series (except silky mouthfeel and sweet taste) or the R2 dilution series (except toasted aroma, woody aroma, sweet taste, and plastic aftertaste). This lack of variation shows the panelists were able to rate the sample attributes across replications consistently.

**Table 2:** Analysis of variance (ANOVA) *F*‐ratios for sensory attributes rated for dilutions of rum 1^a^

Modality	Attribute	Dilution	Panelist	Rep	D × P^b^	D × R^b^	R × P^b^	Adjusted Sample F
Aroma	Alcohol	8.54**	2.16	0.12	0.81	1.33	0.12	
	Caramel	5.67*	4.18*	0.14	1.37	3.26	0.79	
	Maple	4.51*	11.48***	0.21	1.48	2.91	1.23	
	Vanilla	5.64*	5.43**	0.5	1.19	1.51	0.7	
	Dark Fruit	1.26	8.29***	0.07	0.82	0.38	0.53	
	Roasted	6.32*	8.15***	0.07	0.71	0.46	1.19	
	Toasted	2.88	2.87*	0.03	0.59	0.52	0.97	
	Woody	8.15**	6.46**	3.02	1.49	2.77	1.62	
Mouthfeel	Astringent	22.04***	5.59**	0.64	0.82	0.28	1.53	
	Silky	22.41***	6.62**	6.67*	3.94**	2.43	0.73	5.69*
	Slick	14.6***	3.81*	0.97	1.81	0.11	2.1	
	Warming	24.47***	3.09*	0.27	0.62	0.12	0.37	
Taste	Bitter	20.56***	5.9**	2.6	1.23	0.87	1.72	
	Sweet	5.31*	11.47***	5.3*	4.67**	1.68	2.69	1.14
Aftertaste	Bitter	21.35***	7.62***	1.75	0.69	1.19	0.19	
	Brown Spice	11.1**	6.32**	0	0.9	0.54	1.05	
	Vanilla	2.3	3.47*	0.1	1.02	0.44	1.01	1.69
	Plastic	7.22**	3.06*	1.56	4.28**	0.48	2.56	
Aroma‐by‐mouth	Alcohol	36.25***	2.81*	0.06	0.93	0.36	0.35	
	Caramel	7.43**	3.09*	0.04	0.48	0.61	1.29	
	Maple	4.65*	2.88*	2.19	0.53	0.49	0.52	
	Vanilla	3.79*	5.47**	1.51	1.59	1.92	0.59	
	Woody	4.47*	3.29*	4.18	0.72	0.17	0.25	

*, **, *** stand for significance at *p* < .05, *p* < .01, and *p* < .001, respectively.

a F‐ratios are shown as a source of variation.

b D × P, R × P, and D × R represent the interaction between dilution samples and panelists, replications, and panelists, and dilution samples and replications, respectively.

**Table 3:** Analysis of variance (ANOVA) F‐ratios for sensory attributes rated for dilutions of rum 2^a^

Modality	Attribute	Dilution	Panelist	Rep	D × P^b^	D × R^b^	R × P^b^	Adjusted sample F
Aroma	Alcohol	40.85***	8.71***	0.23	2.03	1.22	2.37	
	Caramel	5.94*	11.54***	0.81	1.20	0.15	1.08	
	Maple	3.66	8.94***	1.06	0.94	0.67	2.30	
	Vanilla	5.36*	8.96***	2.64	1.37	1.48	1.10	
	Dark Fruit	3.98*	12.04***	0.16	1.48	0.66	0.80	
	Roasted	2.38	4.15*	0.52	1.42	0.51	1.68	
	Toasted	0.72	15.38***	5.56***	4.18**	1.17	1.58	0.17
	Woody	0.18	9.18***	4.61***	1.94	0.08	1.89	
Mouthfeel	Astringent	15.17***	15.17***	0.24	1.56	0.47	2.18	
	Silky	10.09***	6.85***	1.04	3.19***	2.01	1.18	3.17
	Slick	20.36***	7.36***	0.51	2.96*	4.44*	2.68	6.87**
	Warming	59.21***	11.57***	0.06	1.17	1.20	3.02***	
Taste	Bitter	49.87***	13.75***	0.08	0.78	0.84	6.62***	
	Sweet	3.60	7.58***	6.62*	3.26*	1.00	4.73**	1.11
Aftertaste	Bitter	29.14***	11.98***	2.28	1.11	0.77	2.30	
	Brown Spice	11.79**	5.91**	2.51	1.53	0.01	1.25	
	Vanilla	5.62*	11.8***	0.34	1.80	0.26	1.28	
	Plastic	4.34*	12.72***	6.42*	2.23	3.26	3.38*	
Aroma‐by‐mouth	Alcohol	47.80***	7.54***	2.29	0.57	1.37	0.66	
	Caramel	16.90***	9.47***	0.01	2.08	1.50	0.37	
	Maple	26.65***	17.89***	1.18	3.41*	0.68	3.72*	7.81**
	Vanilla	23.49***	33.16***	1.20	2.58*	0.93	1.57	9.10**
	Woody	1.56	13.48***	0.38	1.77	0.43	0.78	

*, **, *** stand for significance at *p* < .05, *p* < .01, and *p* < .001, respectively.

a F‐ratios are shown as a source of variation.

b D × P, R × P, and D × R represent the interaction between dilution samples and panelists, replications and panelists, and dilution samples and replications, respectively.

Significant panelist variation did exist (*p* < .05) for all attributes for the R1 dilution series (except alcohol aroma) and R2 dilution series. This type of variation is typical of descriptive analysis panels and is most likely a result of panelists not using the entire scale or using different parts of the scale to rate the samples (Lawless & Heymann, 1999a, b; Stone, Sidel, Oliver, Woolsey, & Singleton, 1974). R × P interactions were not significant (*p* > .05) for any attributes in the R1 series nor for most attributes in the R2 series, with the exception of warming mouthfeel, bitter taste, sweet taste, plastic aftertaste, and maple aroma‐by‐mouth. The lack of interaction indicates that panelists were able to agree on the intensity of the attributes in the samples across replications. There were no D × R effects in either series (except for slick mouthfeel in R2 series) indicating that panelists rated the samples similarly across replications.

Significant D × P interactions (*p* < .05) did exist for the R1 series (except for silky mouthfeel, sweet taste, and vanilla aftertaste) and the R2 series (except for toasted aroma, silky mouthfeel, slick mouthfeel, sweet taste, maple aroma‐by‐mouth, and vanilla aroma‐by‐mouth). These interactions indicate that panelists were not able to agree on the order of the intensity of the attributes across samples. Adjusted F‐vales were calculated for attributes that had significant D × R interactions to account for the variation. The adjusted F‐values are shown in Tables 2 and 3. Based on the adjusted *F*‐values and initial *F*‐values for the dilutions, eighteen of the attributes in the R1 series were determined to be significantly different (*p* < .05) including alcohol aroma, caramel aroma, maple aroma, vanilla aroma, roasted aroma, woody aroma, astringent mouthfeel, silky mouthfeel, slick mouthfeel, warming mouthfeel, bitter taste, bitter aftertaste, brown spice aftertaste, alcohol aroma‐by‐mouth, caramel aroma‐by‐mouth, maple aroma‐by‐mouth, vanilla aroma‐by‐mouth, and woody aroma‐by‐mouth. For the R2 dilution series, sixteen attributes were determined to be significantly different (*p* < .05) including alcohol aroma, caramel aroma, vanilla aroma, dark fruit aroma, astringent mouthfeel, slick mouthfeel, warming mouthfeel, bitter taste, bitter aftertaste, brown spice aftertaste, vanilla aftertaste, plastic aftertaste, alcohol aroma‐by‐mouth, caramel aroma‐by‐mouth, maple aroma‐by‐mouth, and vanilla aroma‐by‐mouth. Of the 23 attributes evaluated, all terms (except toasted aroma) were statistically different for at least one of the rum series, indicating that proper attributes were chosen for evaluation.

### 3.1 Effect of dilution on sensory profiles

The dilution of the rum samples, either with water or ethanol (40% ABV), caused significant changes to the sensory profiles of the rums. The results indicated that rum samples diluted with ethanol (R1‐E and R2‐E) had the lowest intensities for all attributes (except silky mouthfeel in the R1 series).

For the R1 dilution series, R1 was significantly different from R1‐E for all attributes, having higher intensities for all attributes except silky mouthfeel (Table 4). Additionally, the two dilutions, R1‐W and R1‐E, significantly differed from each other for most attributes, except for caramel aroma, maple aroma, vanilla aroma, maple aroma‐by‐mouth, and vanilla aroma‐by‐mouth. R1‐W had a higher intensity of all attributes except silky mouthfeel. R1 and R1‐W were not significantly different from each other except for caramel aroma, maple aroma, vanilla aroma, and brown spice aftertaste. Selected significant attribute correlations for the R1 dilution series samples (Table 5) include those between astringent mouthfeel and roasted aroma, warming mouthfeel, and alcohol aroma‐by‐mouth, and a significant negative correlation existed between silky mouthfeel and roasted aroma, astringent mouthfeel, warming mouthfeel, and alcohol aroma‐by‐mouth.

**Table 4:** Mean intensity rating for significant aroma, mouthfeel, taste, aftertaste, and aroma‐by‐mouth attributes of the rum 1 dilution series

Modality	Attributes	R1*	R1‐W*	R1‐E*
Aroma	Alcohol	9.44^A^	7.94^A^	5.19^B^
	Caramel	10.25^A^	8.31^B^	7.56^B^
	Maple	9.63^A^	7.81^B^	7.94^B^
	Vanilla	10.56^A^	8.44^B^	8.50^B^
	Roasted	5.00^A^	4.88^A^	3.19^B^
	Woody	6.38^A^	5.75^A^	4.19^B^
Mouthfeel	Astringent	9.81^A^	9.31^A^	5.56^B^
	Silky	6.63^B^	6.94^B^	10.44^A^
	Slick	8.13^A^	7.00^A^	4.56^B^
	Warming	9.19^A^	9.06^A^	3.81^B^
Taste	Bitter	8.25^A^	8.50^A^	4.69^B^
Aftertaste	Bitter	9.38^A^	9.71^A^	5.69^B^
	Brown Spice	8.38^A^	7.00^B^	5.38^C^
Aroma‐by‐mouth	Alcohol	10.06^A^	9.50^A^	3.81^B^
	Caramel	9.75^A^	8.75^A^	6.94^B^
	Maple	8.75^A^	7.56^A,B^	6.44^B^
	Vanilla	10.19^A^	9.06^A,B^	8.38^B^
	Woody	6.63^A^	6.75^A^	4.75^B^

Superscripts of the same letter within an attribute indicate no significant difference by Fisher's least significant difference (LSD) test at α = .05. “R1” is rum 1, “R1‐W” is 1:2 dilution of rum 1 with water to achieve 20% ABV, “R1‐E” is 1:2 dilution of rum 1 with 40% ethanol to achieve 40% ABV.

**Table 5:** Pearson correlation coefficients for significant attributes for rum 1 dilution series samples

Attributes	Alcohol A	Caramel A	Maple A	Vanilla A	Roasted A	Woody A	Astringent MF	Silky MF	Slick MF	Warming MF
Alcohol_A	1.000									
Caramel_A	0.914	1.000								
Maple_A	0.728	0.944	1.000							
Vanilla_A	0.754	0.956	0.999*	1.000						
Roasted_A	0.956	0.756	0.496	0.530	1.000					
Woody_A	0.997*	0.882	0.676	0.705	0.975	1.000				
Astringent_MF	0.970	0.786	0.537	0.570	0.999*	0.985	1.000			
Silky_MF	−0.960	−0.765	−0.508	−0.541	−1.000**	−0.978	−0.999*	1.000		
Slick_MF	0.999*	0.897	0.699	0.727	0.968	1.000*	0.979	−0.971	1.000	
Warming_MF	0.945	0.730	0.462	0.497	0.999*	0.966	0.996*	−0.999*	0.957	1.000
Bitter_Ta	0.915	0.673	0.390	0.426	0.993	0.942	0.986	−0.991	0.931	0.997
Bitter_AT	0.909	0.662	0.376	0.412	0.991	0.937	0.984	−0.989	0.925	0.995
BrownSpice_AT	0.993	0.956	0.806	0.828	0.914	0.981	0.932	−0.919	0.987	0.898
Alcohol_ABM	0.963	0.770	0.515	0.548	1.000*	0.980	1.000*	−1.000**	0.973	0.998*
Caramel_ABM	1.000**	0.915	0.730	0.756	0.956	0.997	0.969	−0.960	0.999*	0.944
Maple_ABM	0.983	0.973	0.842	0.862	0.886	0.967	0.908	−0.893	0.975	0.868
Vanilla_ABM	0.952	0.994	0.903	0.919	0.821	0.928	0.848	−0.829	0.939	0.799
Woody_ABM	0.917	0.675	0.393	0.429	0.993	0.943	0.987	−0.992	0.932	0.997*

Attributes	Bitter T	Bitter AT	Brown Spice AT	Alcohol ABM	Caramel ABM	Maple ABM	Vanilla ABM	Woody ABM
Alcohol_A								
Caramel_A								
Maple_A								
Vanilla_A								
Roasted_A								
Woody_A								
Astringent_MF								
Silky_MF								
Slick_MF								
Warming_MF								
Bitter_Ta	1							
Bitter_AT	1.000**	1						
BrownSpice_AT	0.85969	0.85177	1					
Alcohol_ABM	0.99025	0.988	0.92246	1				
Caramel_ABM	0.91416	0.90784	0.99296	0.96171	1			
Maple_ABM	0.82546	0.81672	0.998*	0.89604	0.98341	1		
Vanilla_ABM	0.7486	0.73836	0.98224	0.83365	0.9531	0.99219	1	
Woody_ABM	1.000**	1.000*	0.86116	0.99065	0.91532	0.82708	0.7505	1

*, **, *** stand for significance at *p* < 0.05, *p* < 0.01, and *p* < 0.001, respectively.

“A” is aroma, “ABM” is aroma‐by‐mouth, “AT” is aftertaste, “MF” is mouthfeel, “Ta” is taste.

For the R2 dilution series, all attributes were rated higher in R2 compared with R2‐W and R2‐E (Table 6). In addition, all attributes were rated higher in R2‐E than in R2‐W, except for vanilla aroma, vanilla aftertaste, and plastic aftertaste. R2 and R2‐W were significantly different from each other for alcohol aroma, dark fruit aroma, astringent mouthfeel, caramel aroma‐by‐mouth, maple aroma‐by‐mouth, and vanilla aroma‐by‐mouth attributes. Selected significant attribute correlations for the R2 dilution series (Table 7) include those between: bite vs aftertaste, warming mouthfeel vs alcohol aroma‐by‐mouth, slick mouthfeel vs brown spice aftertaste, plastic aftertaste vs alcohol aroma‐by‐mouth, and astringent mouthfeel vs slick mouthfeel, bitter, brown spice, and plastic aftertaste, and alcohol and maple aroma‐by‐mouth.

**Table 6:** Mean intensity rating for significant aroma, mouthfeel, taste, aftertaste, and aroma‐by‐mouth attributes for R2, R2‐W and R2‐E

Modality	Attribute	R2*	R2‐W	R2‐E
Aroma	Alcohol	9.63^A^	7.94^B^	4.88^C^
	Caramel	8.13^A^	7.31^A^	5.81^B^
	Vanilla	8.44^A^	7.50^A,B^	6.19^B^
	Dark Fruit	7.13^A^	5.13^B^	5.50^B^
Mouthfeel	Astringent	10.25^A^	8.81^B^	4.75^C^
	Slick	6.63^A^	5.75^A^	3.13^B^
	Warming	9.63^A^	9.06^A^	3.63^B^
Taste	Bitter	9.06^A^	8.38^A^	4.13^B^
Aftertaste	Bitter	10.25^A^	9.06^A^	6.00^B^
	Brown Spice	7.56^A^	7.13^A^	5.63^B^
	Vanilla	7.06^A^	6.25^A,B^	5.31^B^
	Plastic	4.94^A^	4.50^A,B^	3.44^B^
Aroma‐by‐mouth	Alcohol	10.81^A^	9.38^A^	3.94^B^
	Caramel	8.44^A^	7.06^B^	5.06^C^
	Maple	8.06^A^	7.19^B^	5.38^C^
	Vanilla	8.63^A^	7.19^B^	5.75^C^

Superscripts of the same letter within an attribute indicate no significant difference by Fisher's least significant difference (LSD) test at α = .05.

“R2” is rum 2, “R2‐W” is 1:2 dilution of rum 2 with water to achieve 20% ABV, “R2‐E” is 1:2 dilution of rum 1 with 40% ethanol to achieve 40% ABV.

**Table 7:** Pearson correlation coefficients for significant attributes for R2, R2‐W and R2‐E

Attributes	Alcohol A	Caramel A	Vanilla A	Dark Fruit A	Astringent MF	Slick MF	Warming MF	Bitter T	Bitter AT	BrownSpice AT
Alcohol_A	**1.000**									
Caramel_A	1.000**	**1.000**								
Vanilla_A	0.998*	0.997*	**1.000**							
DarkFruit_A	0.650	0.648	0.702	**1.000**						
Astringent_MF	0.995	0.995	0.985	0.568	**1.000**					
Slick_MF	0.993	0.994	0.983	0.559	1.000**	**1.000**				
Warming_MF	0.963	0.964	0.942	0.421	0.986	0.988	**1.000**			
Bitter_Ta	0.973	0.974	0.955	0.459	0.992	0.993	0.999*	**1.000**		
Bitter_AT	0.996	0.997	0.988	0.584	1.000*	1.000	0.982	0.989	**1.000**	
BrownSpice_AT	0.990	0.990	0.977	0.533	0.999*	1.000*	0.992	0.996	0.998*	**1.000**
Vanilla_AT	0.993	0.992	0.999*	0.738	0.975	0.972	0.923	0.938	0.979	0.965
Plastic_AT	0.998*	0.998*	0.990	0.596	0.999*	0.999*	0.979	0.987	1.000**	0.997*
Alcohol_ABM	0.987	0.988	0.974	0.520	0.998*	0.999*	0.994	0.997*	0.997*	1.000**
Caramel_ABM	0.998*	0.998*	1.000**	0.694	0.987	0.985	0.945	0.958	0.990	0.979
Maple_ABM	0.999*	0.999*	0.994	0.623	0.998*	0.997	0.972	0.981	0.999*	0.994
Vanilla_ABM	0.986	0.986	0.996	0.766	0.964	0.961	0.906	0.923	0.969	0.952

Attributes	Vanilla AT	Plastic AT	Alcohol ABM	Caramel ABM	Maple ABM	Vanilla ABM
Alcohol_A						
Caramel_A						
Vanilla_A						
DarkFruit_A						
Astringent_MF						
Slick_MF						
Warming_MF						
Bitter_Ta						
Bitter_AT						
BrownSpice_AT						
Vanilla_AT	1.000					
Plastic_AT	0.982	1.000				
Alcohol_ABM	0.960	0.996	1.000			
Caramel_ABM	0.998*	0.992	0.976	1.000		
Maple_ABM	0.988	0.999*	0.992	0.996	1.000	
Vanilla_ABM	0.999*	0.973	0.948	0.994	0.980	1.000

*, **, *** stand for significance at *p* < .05, *p* < .01, and *p* < .001, respectively.

“A” is aroma, “ABM” is aroma‐by‐mouth, “AT” is aftertaste, “MF” is mouthfeel, “Ta” is taste.

**Figure 1:**
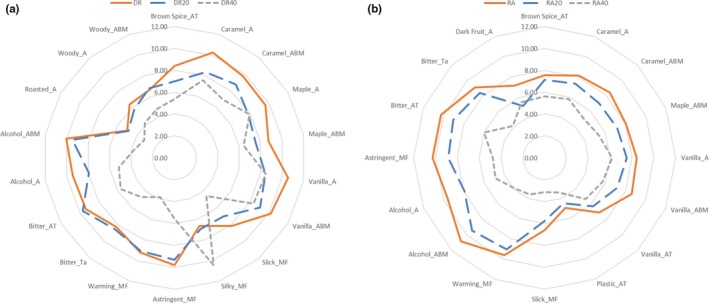
Spider plot of mean significant attribute intensities for (a) R1, R1‐W and R1‐E and (b) R2, R2‐W and R2‐E rum 1. “R1” is rum 1, “R1‐W” is 1:2 (v/v) dilution of rum 1 with water to achieve 20% ABV, “R1‐E” is 1:2 (v/v) dilution of rum 1 with 40% ethanol to achieve 40% ABV. “R2” is rum 2, “R2‐W” is 1:2 dilution (v/v) of rum 2 with water to achieve 20% ABV, “R2‐E” is 1:2 (v/v) dilution of rum 1 with 40% ethanol to achieve 40% ABV. “A” is aroma, “ABM” is aroma‐by‐mouth, “AT” is aftertaste, “MF” is mouthfeel, “Ta” is taste

Interestingly, rums diluted with water possessed nearly the same sensory profiles as the original rums, with only slightly lower intensities for most attributes as shown in the constructed spider plots (Figure 1). In contrast, the results indicate that dilution with 40% ABV profoundly changed the sensory profiles of the rums, especially for the R2 series.

**Figure 2:**
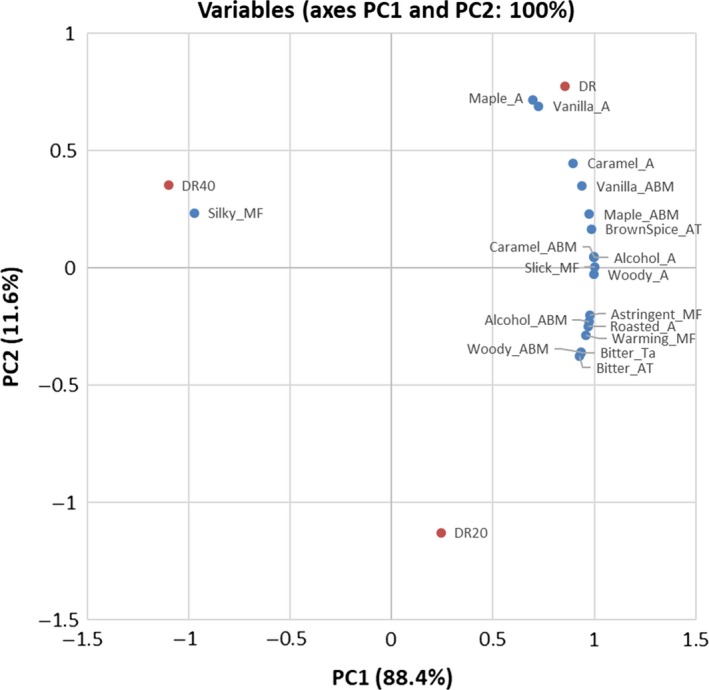
Principal component analysis biplot of significant attributes present on principle component 1 (PC1) and 2 (PC2) by the correlation matrix of mean significant attribute intensity rating across R1, R1‐W, and R1‐E. “R1” is rum 1, “R1‐W” is 1:2 (v/v) dilution of rum 1 with water to achieve 20% ABV, “R1‐E” is 1:2 (v/v) dilution of rum 1 with 40% ethanol to achieve 40% ABV. “A” is aroma, “ABM” is aroma‐by‐mouth, “AT” is aftertaste, “MF” is mouthfeel, “Ta” is taste

**Figure 3:**
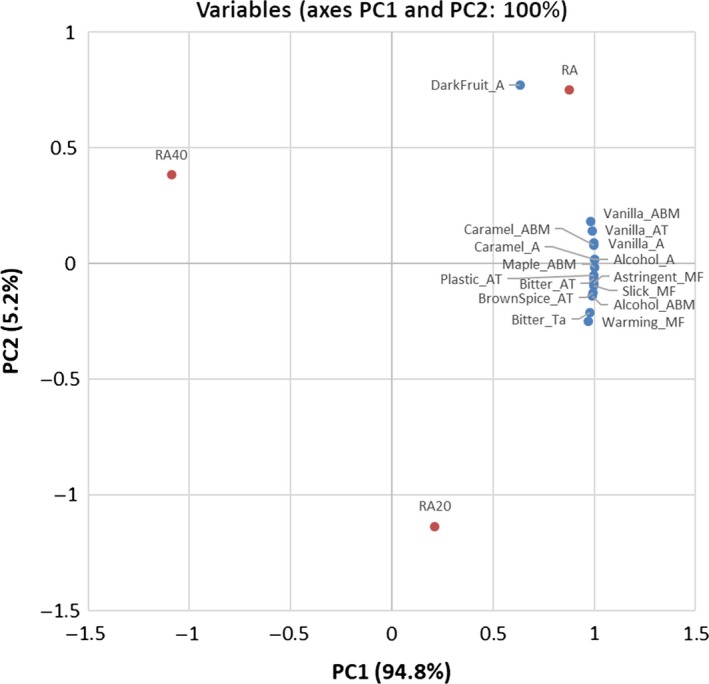
Principal component analysis biplot of significant attributes present on principle component 1 (PC1) and 2 (PC2) by the correlation matrix of mean significant attribute intensity rating across R2, R2‐W, and R2‐E. “R2” is rum 2, “R2‐W” is 1:2 (v/v) dilution of rum 2 with water to achieve 20% ABV, “R2‐E” is 1:2 (v/v) dilution of rum 1 with 40% ethanol to achieve 40% ABV. “A” is aroma, “ABM” is aroma‐by‐mouth, “AT” is aftertaste, “MF” is mouthfeel, “Ta” is taste

Principal component analysis was conducted to reduce the complexity of the data and gain a better visual representation of the results as shown in Figures 2 and 3 (Lawless & Heymann, 1999a,b). The covariance matrix was chosen for sample evaluation as the rums and rum dilutions were scored by a trained panel (Jolliffe, 2014). For the R1 dilution series (Figure 3), the first factor (PC1) contained the majority of the variation between samples (88.4%) and the second factor (PC2) contained the remaining sample variation (11.6%). PC1 contrasts samples high in brown sugar, caramel, maple, vanilla, coconut and chocolate aroma, brown spice and caramel aftertaste, caramel, maple, vanilla and coconut aroma‐by‐mouth, with samples high in alcohol, citrus, and phenolic aroma, warming mouthfeel, and bitter taste. PC2 was mainly defined by slick mouthfeel.

Focusing on the R2 dilution series (Figure 3) the first factor contained the majority of the variation as well (94.8%), with the second factor minimally loaded with the remaining variation (5.2%). PC1 contrasted samples that were high in all significant attributes with those that had lower intensities of those attributes. PC2 was defined by samples differentiated by dark fruit aroma.

These results validate the initial hypothesis that the dilution to a lower alcohol concentration would cause a decrease in attribute intensity, particularly regarding aroma. Previous analytical studies on dynamic systems showed that higher ethanol concentration had higher headspace concentrations of volatiles (Taylor et al., 2010; Tsachaki, Linforth, & Taylor, 2005; Tsachaki et al., 2008). In this study, all aroma attributes were highest in the original rums.

However, the size of the effect between dilutions was not as large as expected, especially since both the alcohol concentration and the flavor compound concentrations were cut in half in the case of R1‐W and R2‐W. No previous work related to the effects of ethanol on flavor perception has considered the dilution effect that occurs when a consumer dilutes the beverage with water. All previous studies have made replicate model solutions that are identical except for their alcohol concentration (Boothroyd et al., 2012; Tsachaki, Linforth, & Taylor, 2009; Tsachaki et al., 2005, 2006, 2008). Even studies that evaluated wine model systems at various ethanol concentrations spiked the solutions with the same concentration of volatiles after the ethanol dilutions were made (Tsachaki et al., 2009). It is likely that the decrease in ethanol concentration caused an increase in the polarity of the system. Additionally, the evaporation effect and subsequent stirring caused by the Marangoni effect and Rayleigh‐Bénard convection (Marangoni, 1865; Rayleigh, 1916) would still occur at 20% ABV, as it has been previously demonstrated in 12% ABV systems (Taylor et al., 2010), and this may explain why the aroma attribute intensities did not decrease as much as expected.

Alternatively, the similar aroma profiles observed between the straight rum and water dilution may be a result of reducing the suppressant effect of ethanol during dilution with water. Ethanol is known to have anesthetic qualities and stimulate trigeminal sensations (Taylor et al., 2010). It is possible that at high ethanol concentrations these qualities may suppress or mask the other odor‐active compounds in distilled beverages. When the ethanol concentration is reduced with water, the suppressant effect of ethanol as an antagonist may diminish. Therefore, in the water dilution, even though the concentration of the congeners is also reduced by half, the release of the suppressant effect of ethanol may compensate for the decrease in volatile concentration, resulting in a similar aroma profile. This is further demonstrated by the dilutions with ethanol. When the concentration and suppressant effects of ethanol were held constant and the concentration of the congeners was cut in half, we observed a significant decrease in the intensity of almost all attributes. More sensory studies are needed to confirm these results and gain a better understanding of how ethanol affects sensory perceptions at higher alcohol levels.

In mouth sensory perceptions, including mouthfeel and taste, also differed as a result of dilution. Regarding mouthfeel, the warming sensation was the same between the original rums and dilutions with water but significantly decreased in the ethanol dilution. This is surprising since previous research has demonstrated that increased ethanol concentration caused a higher rating of hotness or burning mouthfeel sensation (Demiglio & Pickering, 2008; Jones et al., 2008; Nolden & Hayes, 2015). It was expected that the ethanol dilutions would have had the highest warming sensation followed by the original rums and then the dilutions with water. It may be that ethanol, while one factor contributing to the warming sensation of spirits, may not be the only chemical contributing to that perception. These results are interesting as the ethanol used as the reference for warming mouthfeel was the same ethanol used to dilute the samples.

Additionally, previous research demonstrated that increased ethanol concentration causes a decrease in astringency (Demiglio & Pickering, 2008; Fontoin, Saucier, Teissedre, & Glories, 2008). In agreement, R1‐E and R2‐E had the lowest perception of astringency in both series. In the R2 series, the R2‐W was also significantly lower in astringency than R2. This difference could be attributed to the fact that previous studies focused on wines. The high concentration of tannins present in wines may alter the perception of astringency differently than distilled spirits, and in particular as function of ethanol concentration.

Bitter taste was also shown to be significantly lower for the ethanol dilutions in comparison with the original rums and water dilutions. Previous studies have shown increases in ethanol concentration can cause an increase in bitterness (Fontoin et al., 2008; Jones et al., 2008; Nolden & Hayes, 2015), which is contrary to our results. It is possible that other volatile and nonvolatile components dissolved in the rum matrix could account for these differences.

Sweetness was not shown to be significantly different among the rum dilutions; however, previous research has shown that sweetness perception increases with ethanol concentration in wines (Nurgel & Pickering, 2006; Zamora, Goldner, & Galmarini, 2006). It is possible that nonvolatile composition of the two beverages may affect how ethanol concentration impacts sweetness perception.

## 4 CONCLUSION

This was the first study to evaluate the sensory effects of ethanol on distilled spirits. Our results showed that the original rums and dilutions with water were more similar to one another than expected. The samples were only statistically different for several attributes in each series. These results support the age‐old industry tradition of diluting distilled spirits to 20% or 23% ABV for blending and evaluation purposes, and in essence demonstrating that while the intensity of the attributes decreased slightly in the dilutions, the overall flavor profiles were very similar. The results for the ethanol dilutions were not expected, and further research is needed to better understand how ethanol interacts with sensory perceptions at high ethanol concentrations.
